# Involvement of extracellular vesicle microRNA clusters in developing healthy and Rett syndrome brain organoids

**DOI:** 10.1007/s00018-024-05409-7

**Published:** 2024-09-21

**Authors:** Nasim Bahram Sangani, Jarno Koetsier, Ana Rita Gomes, Maria Margarida Diogo, Tiago G. Fernandes, Freek G. Bouwman, Edwin C. M. Mariman, Mehrnaz Ghazvini, Joost Gribnau, Leopold M. G. Curfs, Chris P. Reutelingsperger, Lars M. T. Eijssen

**Affiliations:** 1 Department of Biochemistry, Cardiovascular Research Institute Maastricht (CARIM), Faculty of Health, Medicine and Life Sciences (FHML), Maastricht University, Maastricht, 6200 MD The Netherlands; 2 GKC, Maastricht University Medical Centre, Maastricht, 6229 ER The Netherlands; 3 Department of Bioengineering and iBB-Institute for Bioengineering and Biosciences, Instituto Superior Técnico, Universidade de Lisboa, Lisboa, Portugal; 4 Instituto de Medicina Molecular João Lobo Antunes, Faculdade de Medicina, Universidade de Lisboa, Lisboa, Portugal; 5 Associate Laboratory i4HB – Institute for Health and Bioeconomy, Instituto Superior Técnico, Universidade de Lisboa, Lisbon, Portugal; 6 Department of Human Biology, Institute of Nutrition and Translational Research in Metabolism (NUTRIM), Faculty of Health, Medicine and Life Sciences (FHML), Maastricht University Medical Centre, Maastricht, The Netherlands; 7 Erasmus MC iPS Facility, Erasmus Medical Center, University Medical Center, Rotterdam, Netherlands; 8 Department of Developmental Biology, Erasmus Medical Center, University Medical Center, Rotterdam, Netherlands; 9 Department of Psychiatry and Neuropsychology, School for Mental Health and Neuroscience (MHeNs), Faculty of Health, Medicine and Life Sciences (FHML), Maastricht University, Maastricht, 6200 MD The Netherlands; 10 Department of Bioinformatics—BiGCaT, Institute of Nutrition and Translational Research in Metabolism (NUTRIM), Faculty of Health, Medicine and Life Sciences (FHML), Maastricht University, Maastricht, 6200 MD The Netherlands

**Keywords:** Rett syndrome, Brain organoids, Extracellular vesicles, MicroRNA, chromosome 14 miRNA cluster, Hsa-miR-302/367 cluster

## Abstract

Rett syndrome (RTT) is a neurodevelopmental disorder caused by *de novo* mutations in the *MECP2* gene. Although miRNAs in extracellular vesicles (EVs) have been suggested to play an essential role in several neurological conditions, no prior study has utilized brain organoids to profile EV-derived miRNAs during normal and RTT-affected neuronal development. Here we report the spatiotemporal expression pattern of EV-derived miRNAs in region-specific forebrain organoids generated from female hiPSCs with a MeCP2:R255X mutation and the corresponding isogenic control. EV miRNA and protein expression profiles were characterized at day 0, day 13, day 40, and day 75. Several members of the hsa-miR-302/367 cluster were identified as having a time-dependent expression profile with RTT-specific alterations at the latest developmental stage. Moreover, the miRNA species of the chromosome 14 miRNA cluster (C14MC) exhibited strong upregulation in RTT forebrain organoids irrespective of their spatiotemporal location. Together, our results suggest essential roles of the C14MC and hsa-miR-302/367 clusters in EVs during normal and RTT-associated neurodevelopment, displaying promising prospects as biomarkers for monitoring RTT progression.

**Graphical Abstract:**

**Figure:**
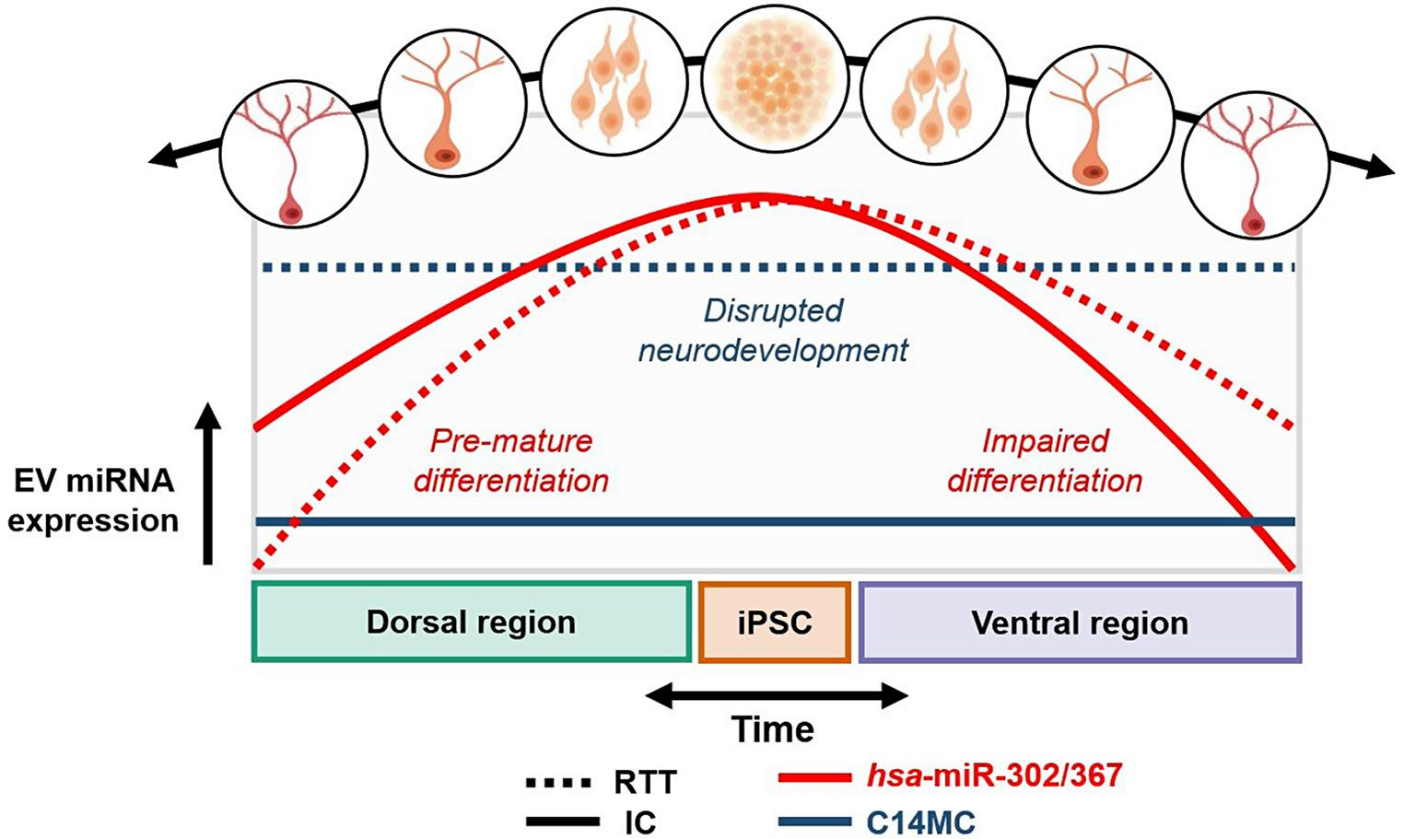

**Supplementary Information:**

The online version contains supplementary material available at 10.1007/s00018-024-05409-7.

## Introduction

Loss-of-function mutations in the X-linked gene *Methyl CpG Binding Protein 2 (MECP2)* are identified as the cause of classical Rett syndrome (RTT) [1]. RTT is a neurodevelopmental disorder characterized by the onset of progressive deterioration of cognitive and motor skills after 6–18 months of age. However, the underlying molecular alterations can arise as early as neural maturation during prenatal brain development [2].

Due to the inaccessibility of living human brain tissue, animal models and in vitro studies have been at the forefront of brain-related research. More recently, the introduction of human brain organoids as an emerging model system has considerably advanced the field [3]. Their unique feature in recapitulating the formation of distinct cortical layers with a diverse neural cell population has provided a better understanding of neurological conditions. This is particularly interesting for neurodevelopmental disorders where dysfunctions arise very early during developmental stages [4]. These spheroid models also reflect the epigenome and gene expression profile of the fetal cortex [5].

In the context of RTT, brain organoids have unraveled new dimensions of the disorder. For instance, Yildirim et al. [6] detected a lesser thickness of the ventricular zones (VZ) region in cerebral organoids derived from RTT patients. This was due to alterations in migration speed and distance, as well as impairment in the neuronal migration pattern. Further impact of *MECP2* mutations on developing organoids was demonstrated in another study by Xiang et al. [7] where cell-type-specific transcriptome analysis revealed severe impairment of cortical interneurons. To investigate brain region-specific alterations in RTT, Gomes et al. [8] applied region-specific brain organoids and found distinct changes in ventral and dorsal forebrain organoids. Specifically, ventral forebrain organoids in RTT, which predominantly includes GABAergic neurons, were characterized by migration defects. Yet, the dorsal forebrain organoids, which mainly include glutamatergic neurons, suffered from premature differentiation.

Brain organoids are also being used to study the role of extracellular vesicles (EVs) in healthy and diseased conditions [9]. These tiny but powerful vesicles enclose heterogeneous contents that efficiently enable them to participate in the regulation of several physiological processes. This includes immune response, cell proliferation and development, tissue repair and regeneration after injury, and cell survival by intracellular transferring of protective factors such as heat shock proteins, which prevent protein aggregation in neurodegenerative disorders [10]. Furthermore, we recently reviewed the physiological role of EVs in the developing brain and indicated their involvement during developmental milestones including neurogenesis, synaptogenesis, synaptic pruning, and myelination [10]. We also highlighted their pathological roles in neurodevelopmental diseases, including RTT [11]. Particularly, considering the mosaicism in females with RTT where not all cells express the mutant allele, non-cell autonomous communication through EVs may allow RTT cells to exert a negative impact on the healthy cells [12, 13].

Amongst the EV content, microRNAs (miRNA, 19–22 nucleotides) are able to silence genes in recipient cells [14]. Using neural cell cultures and animal models, previous studies have suggested a wide range of functions of EV miRNAs in the central nervous system, such as induction of action potential in dorsal root ganglion and communication of the neurogenesis message from differentiated neural progenitor cells to the undifferentiated cells [15, 16]. Furthermore, they have also been shown to regulate dendritic outgrowth, control the integrity of the brain vascular endothelial cells, and modulate glutamate transporter 1 (GLT1) protein expression in astrocytes [17–19].

However, to our knowledge, no prior study has utilized human-derived brain organoids to explore the dynamic miRNA expression profiles in EVs during neuronal development in healthy and diseased conditions. In the present study, we therefore sought to investigate the dynamic role of miRNAs in EVs during normal and RTT-affected neuronal development using *MECP2*-mutant (RTT) and isogenic control (IC) forebrain organoids from patient-derived hiPSCs. The iPSC-derived forebrain organoids harbored the MeCP2:R255X mutation, a common nonsense mutation located within the transcription repression domain - nuclear localization signal (TRD-NLS) region in the MeCP2 protein, that is associated with more severe RTT phenotypes.

## Materials and methods

### hiPSC lines and maintenance

The patient-derived hiPSC cell line, EMC24i/R2 (C6) (46, XX) with a nonsense mutation at R255X with C to T transition (*RTT*) and the respective isogenic (*IC*) cell line EMC24i/R2 (C5) were obtained from Gomes et al. [8]. The ethics approval and written consent for tissue collection were coordinated by Hospital Sant Joan de Déu (HSJD) and Centro Hospitalar de Lisboa Norte (CHLN) Ethics Committees as described by Gomes et al. [8]. Cells were cultured and maintained on Matrigel™ (Corning) - coated plates in serum-free mTeSR^TM^1 Plus medium and passaged every 2–3 days using 0.5 mM EDTA dissociation buffer (Thermofisher Scientific) when reached 85% confluency. iPSC clones were expanded two to three passages before starting the differentiation process.

### Induction of region-specific dorsal and ventral forebrain organoids

To investigate the spatiotemporal alterations in the EV-derived miRNA profile, we adopted the protocol published by Gomes et al. [8] to generate region-specific (i.e., ventral and dorsal) forebrain organoids. The ventral forebrain organoids mimic the anterior side of the forebrain and predominantly contain GABAergic neurons. In contrast, glutamatergic neurons are the main constituent of the dorsal forebrain organoids which replicate the posterior side of the forebrain. These brain regions are suggested to have distinct structural and functional alterations in RTT [8].

**Fig. 1:**
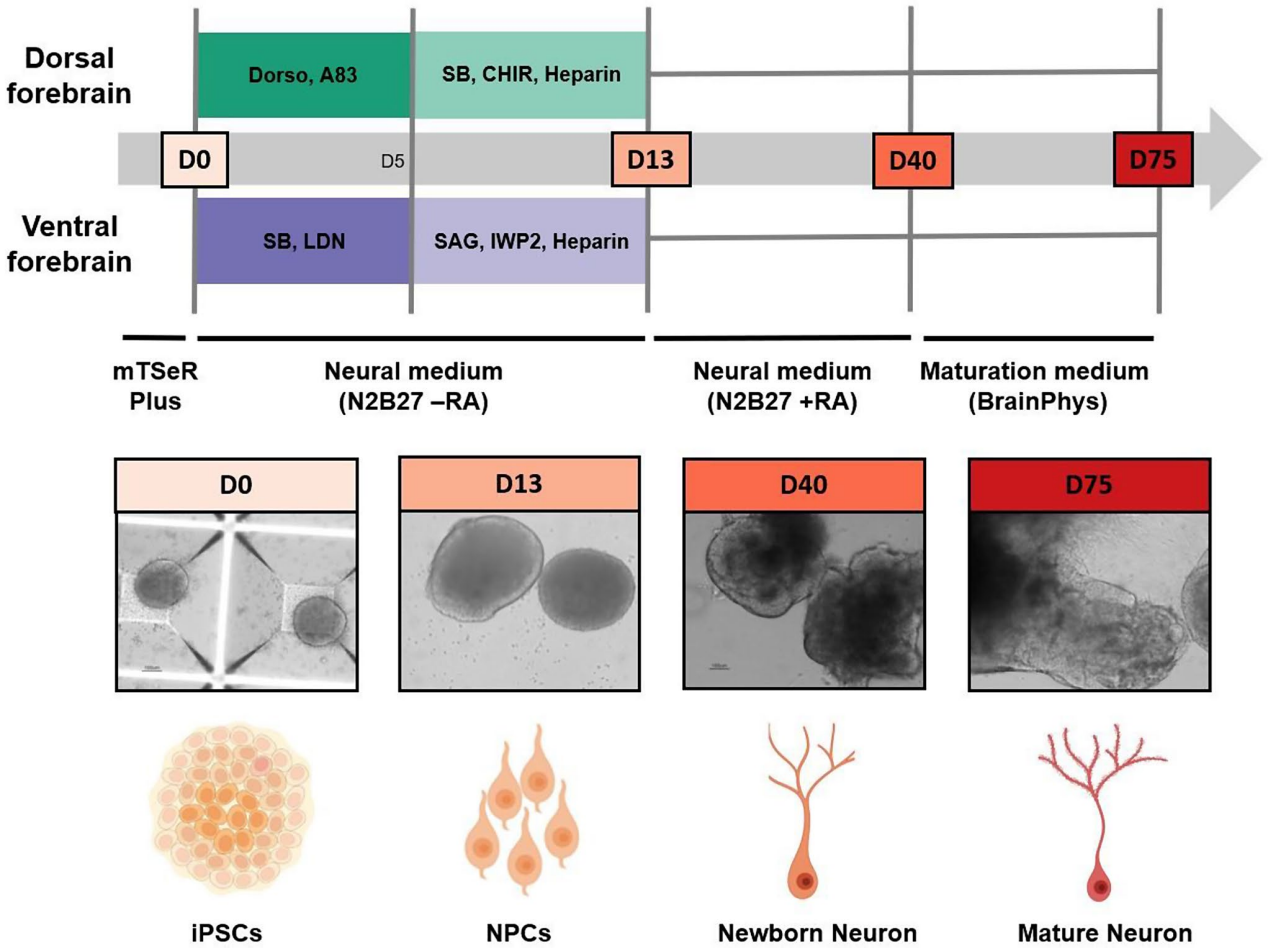
Overview of the applied methodology. Extracellular vesicle miRNA and protein expression data from dorsal and ventral forebrain organoids were collected at four time points, corresponding to the different developmental stages. At day 0 (D0) human iPSCs are seeded in microwell plates to form aggregates. At day 13 (D13) neural progenitor cells (NPCs) as well as neural rosettes appear. At day 40 (D40) of differentiation, newborn neurons of deep cortical layers VI and V emerge. Finally, at day 75 (D75) neurons are mature and fully functional. At this stage, dorsal forebrain cells resemble glutamatergic (excitatory) neurons while cells from the ventral forebrain mimic GABAergic (inhibitory) neurons. Brain organoids also contain astrocytes and glial cells, however, they are not schematically represented here. Photos are representative images taken at each time point. Schematic visualization was created using BioRender.com

As summarized in Fig. 1, we adopted the protocol published by Gomes et al. [8]. In brief, after 30 min of incubation at 37 °C with ROCK inhibitor (ROCKi, Y-27632, 10 µM, StemCell Technologies), hiPSC colonies were dissociated using accutase (Sigma). Cells were then seeded in triplicate (1.5 × 106 cells/well) on microwell plates (AggreWell ^TM^ 800, StemCell Technologies) in mTeSRTM1 Plus supplemented with 10 µM ROCKi for 24 h. The expansion medium was then refreshed entirely without ROCKi supplement. Aggregates usually attain a diameter of 250–300 μm within 2–3 days after which the medium is half changed to induction medium and this day is considered as day 0. The neural induction medium (N2B27) contained 50% of DMEM/F12/N2 (DMEM-F12, (Thermofisher Scientific) supplemented with 1% (v/v) N2 (Thermofisher Scientific), 1.6 g/L Glucose (Sigma), 1% (v/v) PenStrep, and 20 µg/mL Insulin (Sigma) and 50% of Neurobasal/B27 [Neurobasal medium (Thermofisher Scientific) supplemented with 2% (v/v) B27(-Vitamin A)-supplement (Thermofisher Scientific), 2 mM L-glutamine (Thermofisher Scientific) and 1% (v/v) PenStrep. For the dorsal patterned aggregates, the medium was further supplemented with 2 µM Dorsomorphine (Sigma) and 2 µM A83-01 (Tocris) until day 5 with half-medium being refreshed at days 0, 3, and 5. For the ventral forebrain patterning, 10 µM SB-431542 (SB) (Sigma) and 100 nM LDN-193189 (LDN) (Sigma) were added to the medium. At day 5, Cell aggregates (with a population of around 300 aggregates per well of the microwell plate) were transferred to Ultra-Low attachment 6-well plates (Corning). At day 7, the dorsal forebrain culture medium was half changed with fresh medium supplemented with 1 µM CHIR-99021 (tebu-bio), 10 µM SB-431542 (Sigma) and 10 µg/ml Heparin (Sigma) and the ventral forebrain culture medium was half changed with fresh medium supplemented with 2.5 µM IWP2 (Sigma), 100 nM SAG (Millipore) and 10 µg/ml Heparin (Sigma).

### Maintenance and maturation of forebrain organoids

At day 13, the culture medium was half changed with N2B27 (+ Vitamin A) without adding further dorsal and ventral supplemental reagents. This process was continued until day 40 with the medium being changed every 2–3 days. On day 41, the medium was changed to BrainPhysTM Neuronal Medium (StemCell Technologies), supplemented with NeuroCult™ SM1 Neuronal Supplement (StemCell Technologies), N2 Supplement-A (StemCell Technologies), Recombinant Human Brain Derived Neurotrophic Factor (BDNF, PeproTech, 20 ng/mL), Recombinant Human Glial-Derived Neurotrophic Factor (GDNF, PeproTech, 20 ng/mL), dibutyryl cAMP (1 mM, Sigma), and ascorbic acid (200 nM, Sigma). Organoids were maintained for 75 days with every 2–3 days changing one third of the total medium. Supplementary Fig. S1 represents the quality control of differentiation.

### EV extraction and characterization

#### EVs purification

Serum-free conditioned medium was collected from iPSC/organoid cultures that were maintained in vitro for 2 days. Conditioned medium was first centrifuged at 400 ×g for 5 min to remove cells and cell debris. The supernatant was then centrifuged at 2000 ×g for 10 min to purify the medium from apoptotic bodies. The supernatant then underwent differential ultracentrifugation (Beckman, Germany) for 24 min at 10,000 ×g followed by filtration through a 0.22 μm filter (GVA, Millipore). The filtered supernatant was further centrifuged at 100,000 ×g for 47 min. The pellet was resuspended in 1 ml fresh PBS and transferred to MLA-150 tube. To collect the EVs, the final ultracentrifugation was performed using optima™ MAX-XP tabletop ultracentrifuge (Beckman Coulter, USA) for 25 min at 100,000 ×g. All the centrifugation steps were carried out at 4 °C. The pellet was then resuspended in elusion buffer provided within High Pure RNA Isolation Kit (Roche) and the total RNA was extracted and stored at -80 °C until further procedure for transcriptomics. All experiments were performed in triplicates.

#### Nanoparticle tracking analysis (NTA)

To analyze EVs size distribution and concentration, NTA was performed using the ZetaView^®^ nanoparticle tracking analyzer (PMX120, Particle Metrix GmbH, Germany) equipped with software version 8.05.11_SP4. First, the instrument was calibrated with 100 nm polystyrene standard beads with the suggested concentration according to manufacturer’s instruction. EV samples, three replicates for each IC and RTT condition, were diluted to 1 ml in PBS prior to analysis and measured at 11 positions.

### Proteomic profiling

#### Sample preparation

Liquid chromatography–mass spectrometry (LC-MS) was carried out to detect EV protein markers. For EV protein extraction, after the last ultracentrifugation, the pellet was dissolved in Urea buffer (5 M urea, GE Healthcare cat# 17-1319-01, 50 mM ammonium bicarbonate, sigma, cat# 6141). The suspension was snap-frozen in liquid nitrogen, vortexed for 1 min, and underwent 30 min of centrifugation at 18,000×g at 10 °C. Samples were then stored at -80 °C until further procedure for LC-MS.

Protein concentrations were determined by a Bradford based protein assay. 15 µg protein in 50 µl 50 mM ammonium bicarbonate (ABC) with 5 M urea was taken into account. 5 µL of DTT solution (20 mM final) was added and incubated at room temperature for 45 min. The proteins were alkylated by adding 6 µL of iodoacetamide (IAA) solution (40 mM final). The reaction was run at room temperature for 45 min in the darkness. The alkylation was stopped by adding 10 µL of DTT solution (to consume any unreacted IAA) and incubated at room temperature for 45 min. For the digestion 2 µg trypsin/lysC was added to the protein and incubated at 37 ºC for 2 h. 200 µl of 50mM ABC was added to dilute the urea concentration and further incubate at 37 ºC for 18 h. The digestion mix was centrifuged at 2.500 g for 5 min and the supernatant collected for LC-MS.

#### Protein identification using LC-MS

A nanoflow HPLC instrument (Dionex ultimate 3000) was coupled on-line to a Q Exactive (Thermo Scientific) with a nano-electrospray Flex ion source (Proxeon). Of the digest/peptide mixture, 5 µl was loaded onto a C18-reversed phase column (Thermo Scientific Acclaim PepMap C18 column, 75-µm inner diameter x 50 cm, 2-µm particle size). The peptides were separated with a 240 min linear gradient of 4–45% buffer B (80% acetonitrile and 0.08% formic acid) at a flow rate of 300 nL/min.

MS data were acquired using a data-dependent top10 method, dynamically choosing the most abundant precursor ions from the survey scan (250–1250 m/z) in positive mode. Survey scans were acquired at a resolution of 70,000 and a maximum injection time of 100 ms. Dynamic exclusion duration was 30 s. Isolation of precursors was performed with a 2.0 m/z window and a maximum injection time of 200 ms. The resolution for HCD spectra was set to 17,500 and the Normalized collision energy was 30 eV. The under-fill ratio was defined as 1.0%. The instrument was run with peptide recognition mode enabled, but exclusion of singly charged and charge states of more than five.

#### Database search and quantification

The MS data were searched using Proteome Discoverer 2.2 Sequest HT search engine (Thermo Scientific), against the UniProt human database. The false discovery rate (FDR) was set to 0.01 for proteins and peptides, which had to have a minimum length of 6 amino acids. The precursor mass tolerance was set at 10 ppm and the fragment tolerance at 0.02 Da. One miss-cleavage was tolerated, oxidation of methionine was set as a dynamic modification. Carbamidomethylation of cysteines were set as fixed modifications. Label-free quantitation was conducted using the Minora Feature Detector node in the processing step and the Feature Mapper node combined with the Precursor Ions Quantifier node in the consensus step with default settings within Proteome Discoverer 2.2. Normalization was done against the total peptide amount per sample.

### miRNA expression profiling

The generation of miRNA expression data from the extracted total RNA was performed by LC Sciences (Houston, TX, USA) and consisted of the analysis of RNA quality and quantity (*Bioananalyzer 2100*, Agilent, CA, USA), the preparation of small RNA library (*TruSeq Small RNA Sample Prep Kits*, Illumina, San Diego, USA), and single-end sequencing (*Illumina Hiseq 2500*).

Furthermore, the initial data pre-processing included the deletion of low-quality reads, 3’ adapter sequences, and contaminations as well as the removal of other non-coding RNA (rRNA, tRNA, snRNA, and snoRNA) and degradation fragments of mRNA. The remaining sequences were mapped to human miRNA precursors in miRBase 22.0 [20], allowing for length variation at the 3’ and 5’ ends and one mismatch in the aligned sequence (*LC Sciences*,* Houston*,* TX*,* USA*).

Subsequently, the filtering of lowly expressed miRNAs and trimmed mean of M values (TMM) and counts per million (CPM) normalization was performed in-house in accordance with the established edgeR workflow [21]. Boxplots, density plots, and principal component analysis (PCA) plots were constructed for the evaluation of the pre-processed data quality.

### Statistical analysis

#### Characterization of temporal dynamics in miRNA expression

All statistical analyses were performed in the R programming language (v4.2.1). The quasi-likelihood F-test from the edgeR package [21] was used to identify miRNAs that are statistically significantly changed (i.e., false discovery rate (FDR)-adjusted P value < 0.05) between at least two time points in the IC samples. This means that the identified miRNAs are statistically differentially expressed in at least one of the following comparisons: D13 vs. D0; D40 vs. D0; D75 vs. D0; D40 vs. D13; D75 vs. D13; and D75 vs. D40. This was done separately for the samples from the ventral and dorsal regions. These significant miRNAs were subsequently clustered based on their zero centered and unit variance scaled log_2_ CPM value using the Euclidean distance and Ward’s minimum variance linkage method. Besides identifying miRNAs with a dynamic expression profile over time, miRNAs with a ubiquitous expression - defined as a normalized log_2_ CPM value of at least 12 in all IC samples - were identified as well.

#### Identification of miRNA expression alterations in RTT

To identify miRNAs with a differential expression between RTT and IC samples, the quasi-likelihood F-test from the edgeR package [21] was applied at each time point for the two groups, separately for the samples from the dorsal and ventral regions. This means that the expression profiles between RTT and IC was compared at each of the following time points/brain regions: iPSC: D0, Ventral: D13, Ventral: D40, Ventral: D75, Dorsal: D13, Dorsal: D40, and Dorsal: D75. The miRNAs with an FDR-adjusted P value < 0.05 were considered to be significantly differentially expressed at that time point.

#### Pathway analysis

The miRNA-target interaction data from miRTarBase v9.0 [22] was used to get the target genes of all measured miRNAs. Accordingly, using the curated pathway gene sets from WikiPathways (August 10, 2022) [23], overrepresentation analysis (clusterProfiler package [24]) was separately performed on the union of the target genes of ubiquitously expressed miRNAs, the miRNAs with a significantly changed expression between at least two time points, and the miRNAs with a differential expression between RTT and IC samples at any time point. In addition, to reliably estimate the pathways’ significances, 10,000 random miRNA sets with the same set size as the total number of significant miRNAs were used to empirically estimate the distribution of each pathway’s significance under the null hypothesis and to calculate the permutation P value.

## Results

### Characterization of EVs and sample quality

**Fig. 2:**
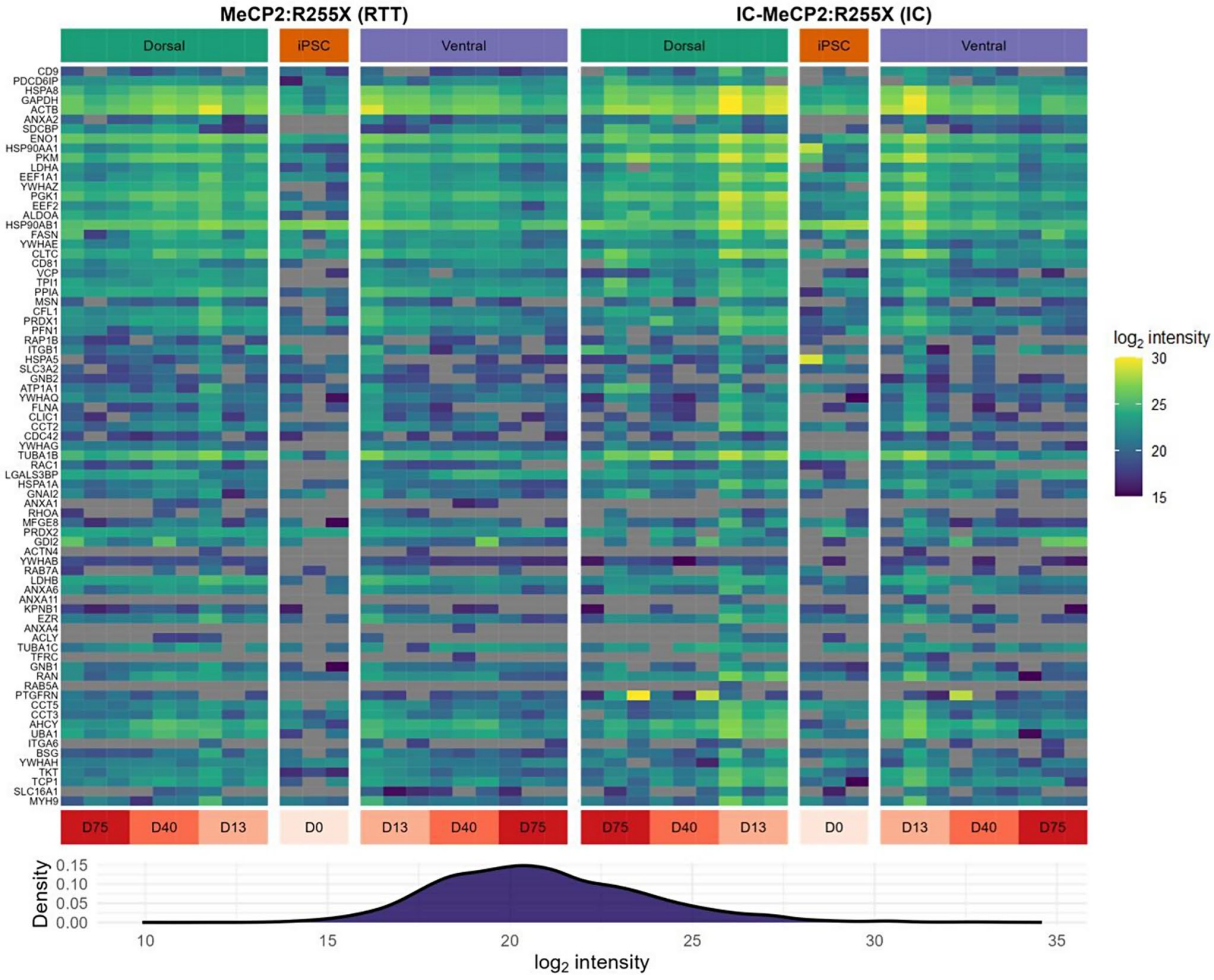
Protein expression of the top 100 ExoCarta exosomal markers. The heatmap shows the normalized log_2_ intensity at different time points (day 0 - day 75) of the top 100 ExoCarta exosomal markers with a detectable expression in at least one sample. The proteins are ordered from top to bottom according to their rank in ExoCarta. The predominantly yellow color on the top of the heatmap indicates that the highly ranked exosomal markers have a high expression in our samples. To give context to the expression level of the exosomal markers, the density plot of the distribution of the log_2_ intensities of all LCMS-identified proteins in all samples is shown at the bottom

The NTA results demonstrated the presence of EVs with a mean diameter of 130 nm for both RTT (MeCP2:R255X) and IC, which is within the range reported for EVs (Supplementary Fig. 2) [25]. Furthermore, the protein expression data was used to evaluate the presence of known exosomal makers in the extracted EVs. As shown in Fig. 2, the top-ranked exosomal protein markers from ExoCarta [26] show high EV expression in our samples. These protein markers include endosomal complexes required for transport (ESCRT)-related proteins such as PDCD6IP, also known as ALIX, HSP90, HSP70 (HSPA8), and tetraspanins including CD9, CD81 as well as RAB proteins. Finally, the quality control plots of the miRNA expression data demonstrate good quality and are provided in Supplementary Figs. 3–4.

### Temporal dynamics of extracellular vesicle miRNA expression

**Fig. 3:**
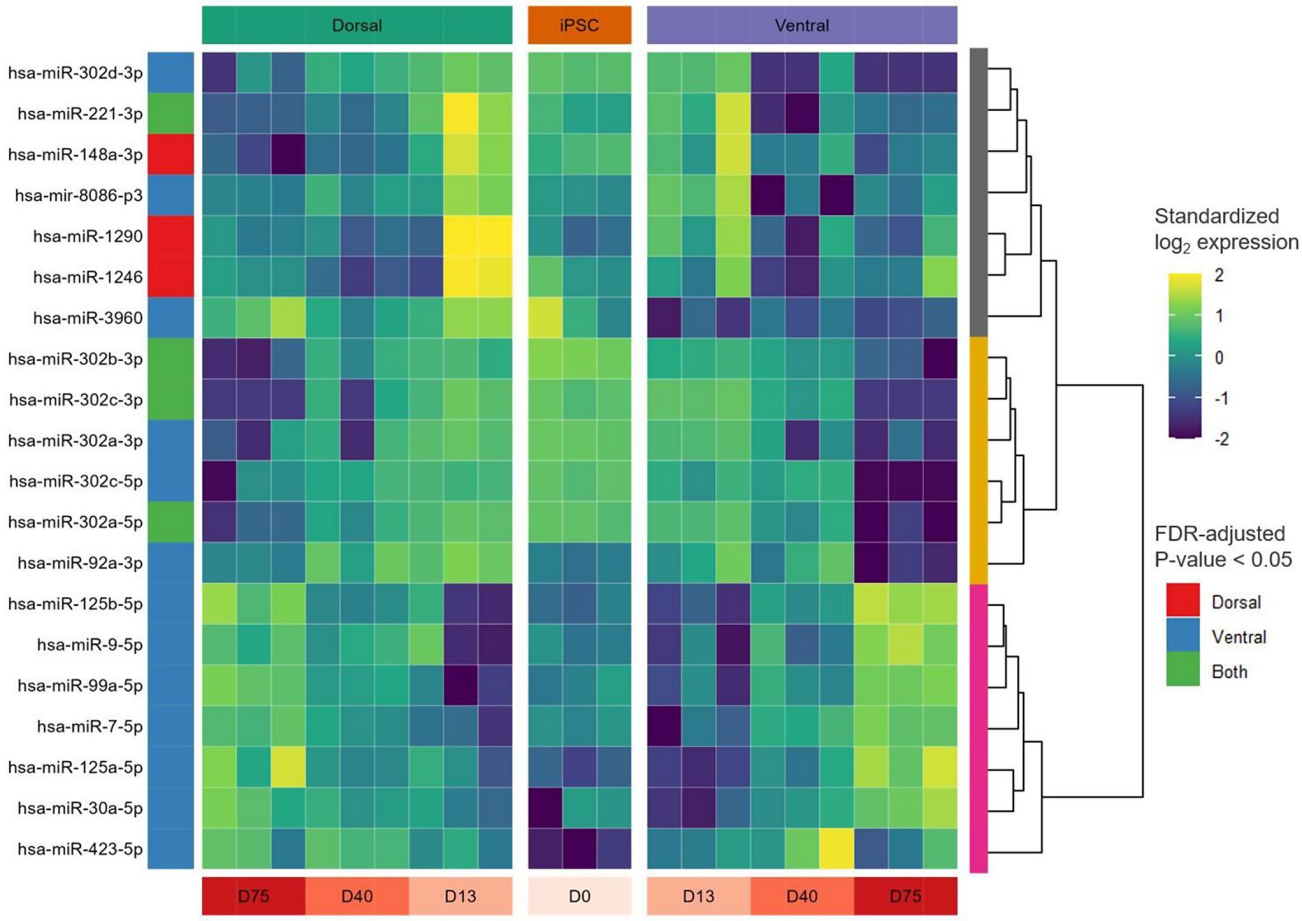
miRNAs with a dynamic temporal expression profile in IC samples. The heatmap shows the standardized (zero centered) expression (log2 CPM) of 20 miRNAs (vertical axis) for each replicate per region and time point (horizontal axis) in the IC samples. The shown miRNAs are significantly differentially expressed (FDR-adjusted P value < 0.05) between at least two time points in the ventral and/or dorsal region (see color bar on the left). The yellow and pink clusters include miRNAs with a, respectively, decreasing and increasing expression over time, while the gray cluster encompasses the remaining miRNAs

To evaluate the changes in miRNA expression during normal brain development, statistical analysis was performed to identify the miRNAs with a dynamic temporal expression profile. These dynamic miRNAs were defined as having an altered expression between at least two time points in the ventral and/or dorsal IC forebrain organoids. From this statistical analysis, twenty miRNAs were found to be differentially expressed. The hierarchical clustering of these miRNAs resulted in three miRNA clusters with distinct temporal expression profiles (Fig. 3).

WikiPathway overrepresentation analysis on the yellow and pink clusters - which encompass miRNAs with a, respectively, decreasing and increasing expression over time - did not yield any significant pathways after FDR adjustment (Supplementary Tables S1–S2). However, the *“Notch Signaling Pathway (WP61)”* was the most significantly enriched pathway of the yellow cluster (permutation P value = 5e-4), while three PI3K/AKT- and JAK/STAT-signaling-related pathways (i.e.,* “EGFR tyrosine kinase inhibitor resistance (WP4806)”*, *“Prolactin signaling pathway (WP2037)”*, and *“Leptin-insulin signaling overlap (WP3935)”)* formed the topmost significantly enriched pathways of the pink cluster (permutation P value = 3e-4, for all three pathways). Notably, *AKT1* is the predominant target gene of these dynamically expressed miRNAs, targeted by three miRNAs from both the yellow (i.e., hsa-miR-302a-3p, -302b-3p, and -302c-3p) and the pink (i.e., hsa-miR-125b-5p, -99a-5p, and -125a-5p) cluster. In contrast to the pink and yellow clusters, the gray cluster does not have a clearly distinctive miRNA expression profile and contains several miRNAs with different temporal dynamics in the ventral and dorsal regions.

Furthermore, ten ubiquitously expressed miRNAs with a log_2_ CPM of at least 12 among all IC samples were identified as well (Supplementary Fig. S5). The WikiPathways overrepresentation analysis on this gene set yielded no significant results after FDR adjustment (Supplementary Table S3).

### Decreasing expression over time of the hsa-miR-302/367 cluster

**Fig. 4:**
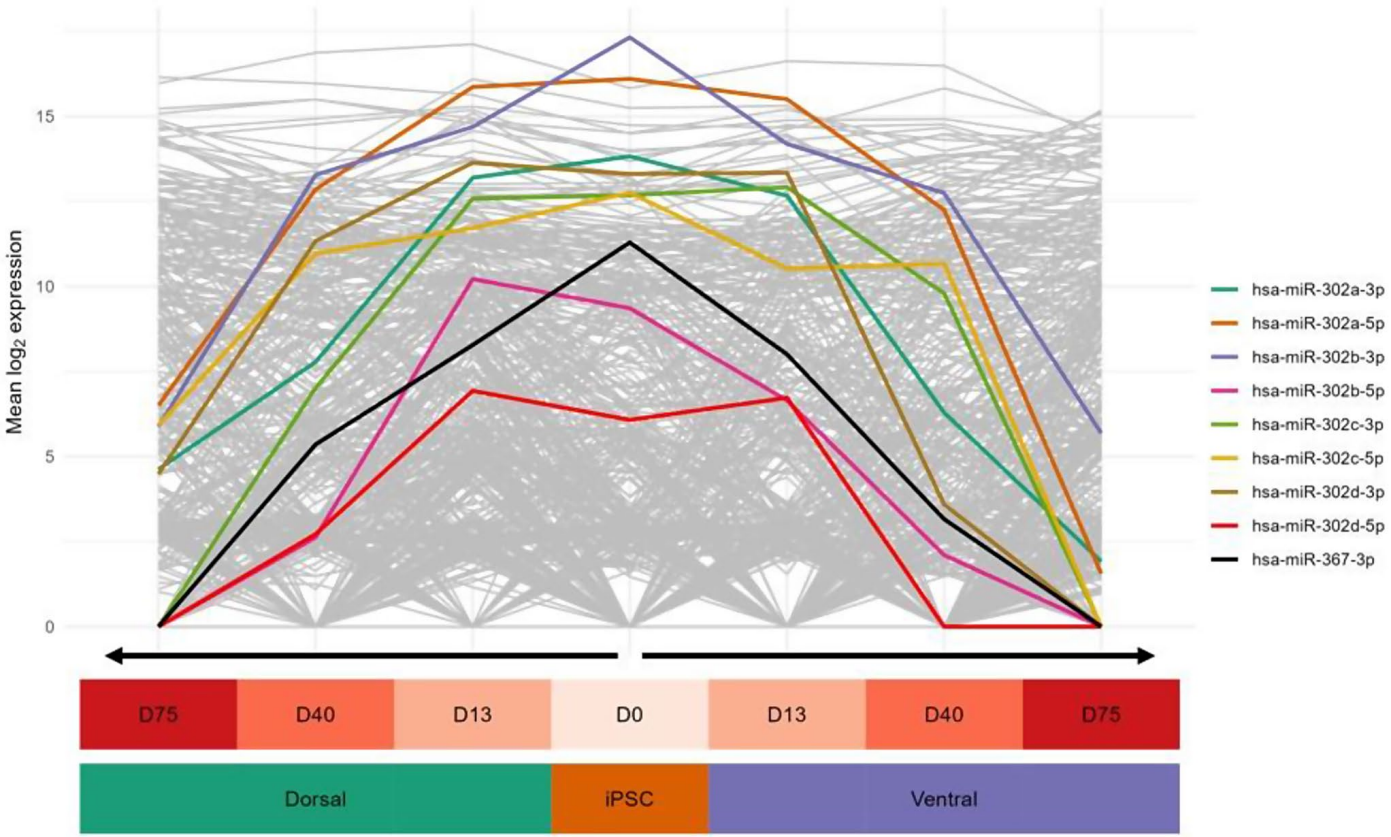
Expression of the hsa-miR-302/367 cluster over time in IC samples. The mean expression (log_2_ CPM) per time point and region is shown for all members of the hsa-miR-302/367 cluster in the IC samples. All members show a decreasing expression over time. The gray lines in the background are the expression patterns of all other miRNAs

**Fig. 5:**
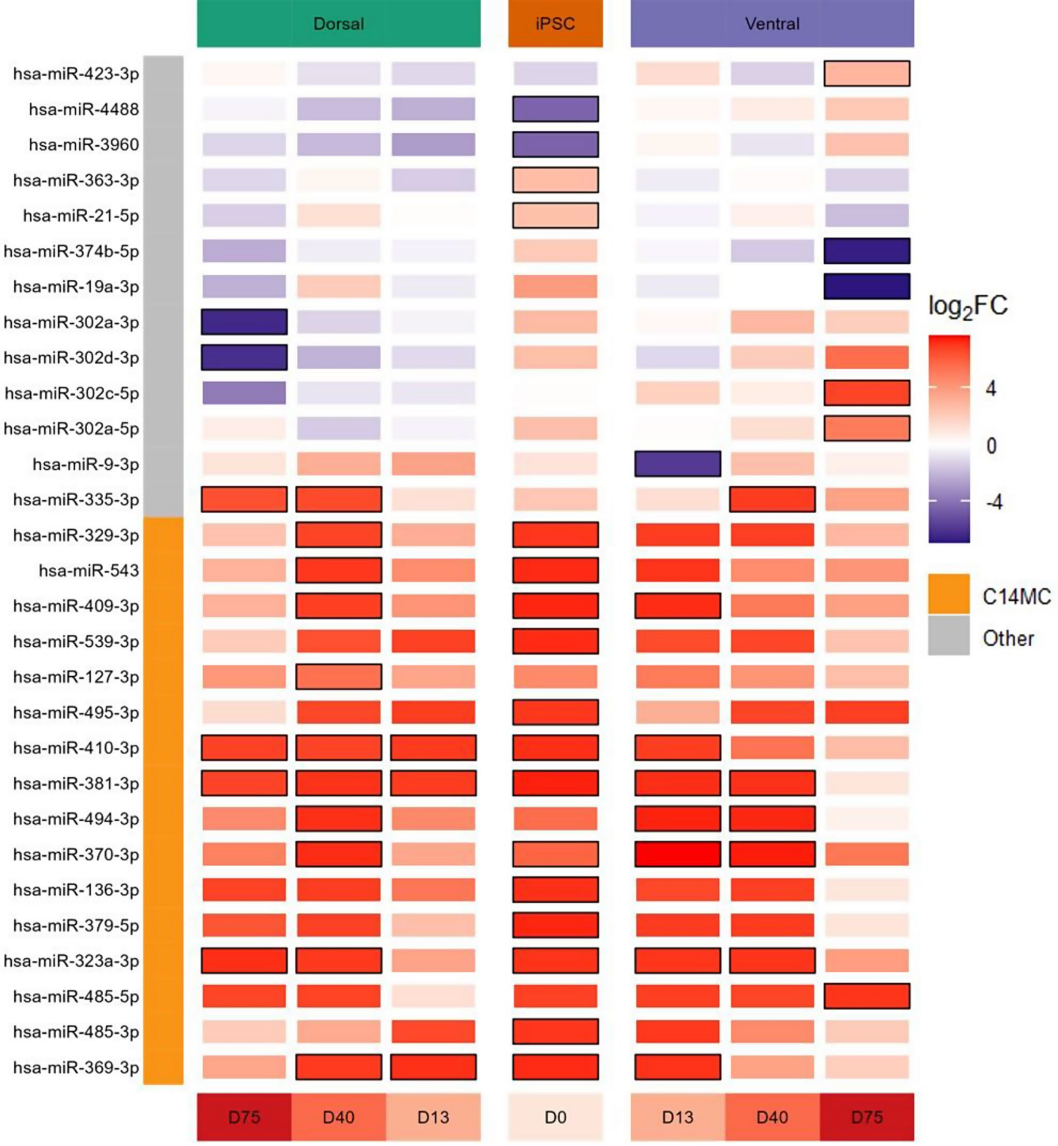
Heatmap of differentially expressed miRNAs. The log_2_FC per time point and region are shown for the miRNAs with a significantly different expression between RTT (MeCP2:R255X) and IC in at least one time point. The black border indicates statistical significance (i.e., FDR-adjusted P value < 0.05). The color bar on the left indicates whether the corresponding miRNA is part of the chromosome 14 miRNA cluster (C14MC)

From the twenty miRNAs with a significantly different expression over time in the IC samples (Fig. 3), six species belong to the hsa-miR-302/367 cluster located on chromosome 4 (4q25 region). Noteworthy, all members of this cluster have a highly correlated expression profile, exhibiting a decreasing expression over time in both dorsal and ventral regions of the IC samples (Fig. 4). Furthermore, when comparing the expression of the IC and RTT forebrain organoids, four members of the hsa-miR-302/367 cluster were found to be significantly up- or downregulated at day 75 in the dorsal or ventral region of RTT forebrain organoids (Fig. 5).

### Elevated expression of the chromosome 14 miRNA cluster in RTT

**Fig. 6:**
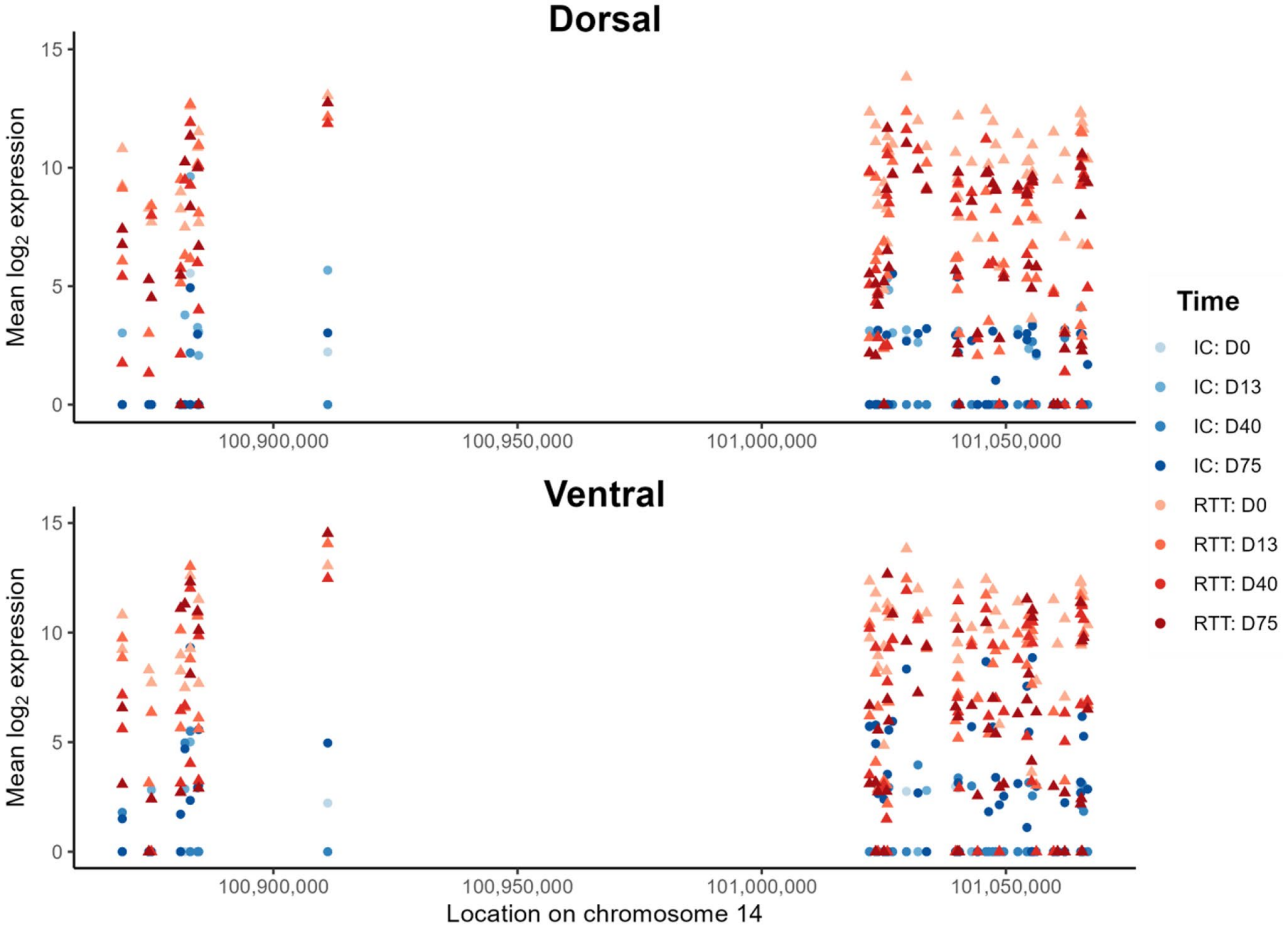
Expression of miRNAs from the chromosome 14 miRNA cluster (C14MC). Mean expression per time point and region is shown for the genomic location of members of the C14MC (*genome build: GRCh38*). There is an overall elevated expression among the C14MC members in the RTT (MeCP2:R255X) samples

For the ventral and dorsal forebrain organoids, the EV-derived expression profile was compared between RTT and IC at each timepoint (i.e., D0, D13, D40, and D75). As shown in Fig. 5, twenty-nine miRNAs were differentially expressed between RTT and IC samples in at least one time point of the ventral or dorsal organoids. WikiPathways overrepresentation analysis on the set of differentially expressed miRNAs did not yield any significant results after FDR-adjustment (Supplementary Table S4). However, the *“Serotonin HTR1 group and FOS pathway (WP722)”* was the most significantly enriched pathway with a permutation P value of 1.6e-3. In this pathway, *CREB1* and several protein kinases (i.e., *ELK4*, *MAP3K1*, *MAPK1*, and *RPS6KA5*) are targeted by at least four differentially expressed miRNAs (Supplementary Fig. S6). Interestingly, more than half of the differentially expressed miRNAs belong to the chromosome 14 miRNA cluster (C14MC) located at the chr14q32 region. Although not all members of this cluster reached statistical significance, an overall higher expression in the RTT samples across the entire cluster is seen in Fig. 6.

## Discussion

In the present study, we characterized the time-dependent EV expression profiles in region-specific MeCP2:R255X RTT and IC forebrain organoids generated from patient-derived iPSCs. Hereby, we identified several miRNA species and clusters that potentially play a key role in normal and RTT-affected neuronal development.

### Increasing EV expression of differentiation-associated miRNAs during neuronal development

We found several miRNAs with an increasing EV expression profile during the progression of neuronal development (pink cluster, Fig. 3) which have before been identified as key players in neuronal differentiation. For instance, hsa-miR-125 and hsa-miR-9 have previously been suggested to promote the differentiation of iPSCs to neural stem cells [27]. In addition, hsa-miR-99a, another miRNA with an increasing EV expression, has also been shown to be upregulated during in vitro neuronal differentiation [28]. Furthermore, Peng et al. [29] and Li et al. [30] demonstrated the anti-proliferative potential of hsa-miR-30a and hsa-miR-7-5p, respectively, in glioma (stem) cells. The top three pathways enriched with the target genes of these miRNAs with an increasing EV expression over time (pink cluster, Fig. 3), all include PI3K/AKT- and JAK/STAT-signaling as part of their diagrams, signaling pathways that have previously been shown to promote neurogenesis and synaptic plasticity [31–33]. Together, the miRNAs with an increasing EV expression over time may be anti-proliferative and promote the differentiation of recipient cells into mature neurons.

### Decreasing EV expression of the hsa-miR-302/367 cluster during neuronal development

Additionally, in the IC samples, members of the hsa-miR-302/367 cluster were found to have a decreasing EV expression during the progression of neuronal development (Figs. 3 and 4). This miRNA cluster is located on human chromosome 4 within the intron of the long non-coding gene *MIR302CHG* and the coding gene *LARP7* in the sense and antisense direction, respectively [34]. Notably, hsa-miR-302a, -302b, -302c, and -302d are highly homologous and share most of their target genes [35]. Genetic variants in the host gene of the hsa-miR-302/367 cluster have been associated with Alazami Syndrome, a disorder characterized by growth restriction and intellectual disability, suggesting an important role of this genomic region in (neural) development [36]. Indeed, the hsa-miR-302/367 cluster has previously been shown to control ectodermal differentiation and to be necessary for the maintenance of pluripotency of iPSCs [37, 38]. Furthermore, Kulcenty et al. [27] showed that, compared to iPSCs, hsa-miR-302 is downregulated in neural stem cells. Besides the hsa-miR-302/367 cluster, hsa-miR-92a, which is another known promoter of cellular proliferation [39], also showed a decreasing expression over time (Fig. 3). In the present study, the high EV expression of these miRNAs in the early stages of neuronal development might thus promote proliferation and the maintenance of pluripotency of neighboring cells. Interestingly, the Notch signaling pathway was found to be the most significantly enriched pathway by miRNAs with a decreasing EV expression over time (yellow cluster, Fig. 3), indicating that the modulation of this pathway by EV miRNAs is predominantly active during the early stages of neuronal development. Indeed, by regulating both neuronal differentiation and proliferation, the Notch signaling pathway is known to be a key regulatory pathway during early neurogenesis [40], emphasizing the need for an active miRNA-dependent regulation during these early stages.

### Region-specific EV expression alterations of the *hsa*-miR-302/367 cluster in RTT

Besides a decreasing expression over time during normal neuronal development, we found a significantly lower EV expression of two members of the hsa-miR-302/367 cluster after 75 days in the dorsal region of the RTT brain organoids as compared to their isogenic controls (Fig. 5). As the hsa-miR-302/367 cluster is required for the maintenance of pluripotency, the downregulation of these miRNAs may lead to the early differentiation of neuronal cells in the dorsal region. In line with our findings, through immunocytochemistry and flow cytometry analyses, Gomes et al. [8] also identified premature differentiation in the dorsal region of RTT brain organoids. In contrast, after 75 days in the ventral region, we found two members of the hsa-miR-302/367 cluster with a significantly upregulated EV expression in RTT brain organoids (Fig. 5), which may inhibit late neuronal differentiation in the ventral region. As cellular differentiation is prerequisite for neuronal migration [41], the upregulated EV expression of the hsa-miR-302/367 miRNA cluster might be partially responsible for the impaired neuronal migration in the ventral region of RTT brain organoids as observed by Gomes et al. [8]. Interestingly, Mellios et al. [42] found an upregulation of hsa-miR-302a-3p in cultured neurons and neural progenitors with, just as our hiPSC cell line, a *MECP2* mutation in the transcription repression domain (TRB), further emphasizing the importance of this miRNA cluster in RTT pathogenesis.

### Elevated EV expression of C14MC in RTT

Besides the hsa-miR-302/367 cluster, our results also suggest an important role of the C14MC in RTT pathology. Specifically, an overall higher expression in RTT versus IC of most members of this cluster in RTT brain organoids was observed, with 30% of the miRNAs (15 out of 50) reaching statistical significance (Figs. 5 and 6). C14MC is one of the largest clusters of miRNAs in the human genome located at the imprinted *DLK1-DIO3* domain on the long arm of chromosome 14 (14q32) [43]. C14MC has been considered as a pregnancy-related cluster with a crucial role in placental development. The miRNAs of this cluster are implicated in the regulation of various cellular processes, including proliferation, differentiation, and apoptosis. Their dysregulations have also been reported in various cancer types including neuroblastoma and gliomas [43].

In our findings, the differentially expressed miRNAs, including several C14MC members, were found to target serotonin receptor 1 signaling (i.e., Serotonin HTR1 group and FOS pathway, WP722, Supplementary Fig. S6). The upregulation of C14MC in RTT may thereby cause excessive inhibition of this signaling pathway. In line with these findings, treatment that enhances serotonin signaling has previously been shown to improve RTT symptoms [44, 45]. Besides their role in serotonin signaling, the miRNAs of the C14MC regulate a set of genes critical for growth and brain development. For instance, miR-329-3p was shown to regulate neural stem cell proliferation by inhibiting E2F1 expression [46]. Another member of the cluster, miR-409-3p, was shown to have a functional role in refining neuronal cell fate within neocortical layer 5 by controlling intermediate neural progenitor cell (IPC) proliferation [47]. MiR-409-3p was further shown to support the corticospinal projection identity of neurons [48]. The regulatory effect of miRNAs in C14MC on neurogenesis can be further expanded to other members. For instance, Rago et al. [49] revealed the involvement of the murine homolog of human C14MC (miR-379/410 cluster) in the regulation of proliferation and differentiation of neural progenitor cells and newborn migrating neurons. Moreover, the function of this miRNA cluster has also been shown to be essential for activity-dependent dendritic outgrowth in hippocampal neurons [50].

Regulation of synaptic transmission is another area where C14MC is actively involved. For example, the murine C14MC homolog plays a role in controlling the expression of genes related to synaptic activity and neuron function [51] and two members of the C14MC, miR-329-3p and miR-495-3p, have been reported to regulate homeostatic synaptic depression (HSD) in excitatory neurons through inhibition to ensure the protection of excitatory neurons from over-excitation [52].

The activities of miRNAs within C14MC also ensure neuroprotection and confer anti-inflammatory effects. Particularly, overexpression of miR-410-3p, a member of the C14MC, has a preventive effect against apoptosis and promotes neuronal survival in hypoxic-ischemic brain damage (HIBD) models [53, 54]; possibly such compensatory mechanism is also acting in RTT neurons. Furthermore, overexpression of this miRNA was able to inhibit anesthesia-induced hippocampal neuron apoptosis [55]. Other members of the C14MC, including miR-379-5p [56], miR-381-3p [57], and miR-494-3p [58, 59], have also been demonstrated to have neuroprotective and/or anti-inflammatory roles in the central nervous system. Finally, studies have reported the involvement of miR-485, a well-studied member of the C14MC, in peripheral nerve regeneration [60], neuronal survival [61], regulation of neurite outgrowth, axonal development, and synapse formation [62], reduction of seizure frequency and the number of epileptiform spikes [63], and apoptosis of glioblastoma cells [64].

### Known involvement of C14MC in neuropathophysiology

Apart from its role during brain development, C14MC is involved in the pathology of several other neurological diseases, including Alzheimer’s disease (AD), Parkinson’s disease (PD), and amyotrophic lateral sclerosis (ALS). Among the individual miRNAs within the cluster, miR-485 is the most well-studied with implications in both AD and PD as described in more detail by Ryu et al. [65]. Interestingly, the overexpression of miR-485-5p have also been observed in the exosomes isolated from cerebrospinal fluid (CSF) of patients with AD and PD [66]. Both mature miRNAs produced from miR-485 are significantly upregulated in our results pointing to the possible existence of common disrupted pathway(s) between RTT and other neurological diseases.

MiR-494-3p is another miRNA of the cluster with implications in PD. Research by Geng et al. [67] indicated that upregulation of miR-494-3p exacerbates motor impairment in a PD mouse model by targeting SIRT3. There are also miRNAs of the cluster detected in ALS; Capauto et al. [68] discovered a crosstalk between Gria2, miR-409, and miR-495 in mouse embryonic stem cell-derived motor neurons with a mutation in the *FUS* gene. They verified a circuitry in which miR-409 and miR-495 downregulate Gria2, a subunit of the glutamate AMPA receptor, which has an essential role in excitatory neurotransmission.

One of the mechanisms by which a MeCP2 loss-of-function mutation can potentially lead to the overexpression of the C14MC was explored by Wu et al. [69], who identified the upregulation of 22 miRNA species from the mouse homolog of the C14MC (i.e., *Dlk2-Gtl2* imprinting domain) in *Mecp2*-knockout mice. Additional analyses by the authors revealed several MeCP2 binding sites within this genomic region and an upregulation of histone H3/H4 acetylation in *Mecp2*-knockout mice, suggesting that MeCP2 can directly regulate the expression of miRNAs from the C14MC. In line with our results, their findings indicate that MeCP2 loss-of-function enhances C14MC expression. A possible indirect mechanism by which MeCP2 can regulate C14MC expression might relate to its reported negative regulation of the *Mef2c* gene in mice [70], a gene that was shown to promote the expression of multiple miRNAs from the mouse homolog of the C14MC [50]. Hence, a loss-of-function mutation in the *MECP2* gene might lead to the upregulation of *MEF2C* expression, which in turn may promote C14MC transcriptional activity.

### Strengths, limitations, and future directions

A major strength of our study is the use of region-specific brain organoids, which - by mimicking an in vivo human forebrain - is a more realistic model of the human brain compared to (fibroblast or neuronal) cell cultures as well as a good alternative to animal models. The use of brain organoids, therefore, allowed us to gain novel insights into the molecular pathophysiology of RTT. Novel insights also came from the measurements at multiple developmental stages, which enabled us to investigate the temporal dynamics of EV miRNA expression in normal and RTT-associated brain development, something that to our knowledge has never been done before. Finally, the use of isogenic controls - which share the same genetic background as the *MECP2* mutant brain organoids - allowed us to confidently attribute the found alterations in the EV miRNA expression profile to the mutated *MECP2* gene. However, prior studies demonstrated distinct molecular phenotypic characteristics for different *MECP2* mutations [8, 42], raising the possibility that some of the identified molecular alterations might only occur for the MeCP2:R255X mutation in combination with the patient-specific genetic background. Although the current findings are mostly supported by the literature, additional investigations are needed to validate the identified molecular alterations in other patients with different genetic backgrounds and RTT-causing *MECP2* mutations.

Since EVs are known to be able to cross the blood-brain barrier [71], the validated RTT-specific EV miRNAs might, in the future, be used as blood-based biomarkers for monitoring the progression of RTT after, for example, a specific intervention strategy. EV-derived biomarkers have already been suggested to be promising diagnostic markers for various neurodegenerative disorders, such as AD and PD [72], and might thus as well be promising for RTT. Specifically, miRNA species from the C14MC could potentially serve as such EV biomarkers. The use of freely circulating miRNAs as biomarkers for RTT has been previously suggested by Sheinerman et al. [73], who reported several, including the miR-323-3p species from C14MC, as biomarkers for RTT progression and treatment response [73]. Furthermore, many C14MC members have also been suggested as potential blood- or CSF-derived biomarkers for several other neurological disorders. For instance, miR-127-3p has been demonstrated to be a promising diagnostic marker for ALS [74], glioblastoma [75], and frontotemporal dementia [76], while the C14MC species miR-136-3p [77], miR-495-3p [77], and miR-543 [78] have been suggested as potential biomarkers for PD diagnosis. After having validated the alterations in the EV expression of C14MC species in other RTT patients, future research is also needed to investigate whether the (validated) alterations are also observed in blood- or CSF- derived EVs.

In summary, we used hiPSC-derived forebrain organoids to characterize the EV miRNA expression profiles during normal and RTT-associated neuronal development. Hereby, we emphasized the importance of the EV expression of the hsa-miR-302/367 cluster during CNS development. Furthermore, we are also the first to report alterations in the EV expression of the C14MC miRNA species in RTT. Future studies could aim at validating our findings for other RTT-causing *MECP2* mutations. Once validated, the C14MC miRNAs from biofluid-derived neuronal EVs have the potential to be used as biomarkers for monitoring RTT progression and the effect of intervention strategies.

## Electronic supplementary material

Below is the link to the electronic supplementary material.

Supplementary Material 1

## Data Availability

The data that support the findings of this study are available from the corresponding author upon reasonable request.

## Abbreviations

AD: Alzheimer’s Disease
ALS: Amyotrophic Lateral Sclerosis
C14MC: Chromosome 14 MiRNA Cluster
CPM: Counts Per Million
CSF: Cerebrospinal Fluid
DTT: Dithiothreitol
EV: Extracellular Vesicle
FDR: False Discovery Rate
hiPSC: Human Induced Pluripotent Stem Cell
IAA: Iodoacetamide
IC: Isogenic Control
LC-MS: Liquid chromatography–mass spectrometry
log_2_FC: log_2_ Fold Change
MECP2: Methyl CpG Binding Protein 2
microRNA: miRNA
NPC: Neural Progenitor Cell
NTA: Nanoparticle Tracking Analysis
PCA: Principal Component Analysis
PD: Parkinson’s Disease
RTT: Rett Syndrome
