# Supplementary material for: Involvement of extracellular vesicle microRNA clusters in developing healthy and Rett syndrome brain organoids
Source: Cell Mol Life Sci. 2024 Sep 21;81(1):410. doi: 10.1007/s00018-024-05409-7 (PMC11416455; doi:10.1007/s00018-024-05409-7)
Supplement: Supplementary file 1 — Supplementary Material 1 [file 18_2024_5409_MOESM1_ESM.pdf]

Supplementary Materials

Involvement of extracellular vesicle microRNA clusters in developing healthy and Rett Syndrome brain organoids

Nasim Bahram Sangani[1,2], Jarno Koetsier[1,2], Ana Rita Gomes[3,4,5], Maria Margarida Diogo[3,5], Tiago G. Fernandes[3,5], Freek G. Bouwman[6], Edwin C. M. Mariman[6], Mehrnaz Ghazvini[7], Joost Gribnau[7,8], Leopold M. G. Curfs[2], Chris P. Reutelingsperger[1,2,*], Lars M.T. Eijssen[9,10]

1 Department of Biochemistry, Maastricht University, Cardiovascular Research Institute Maastricht, 6200 MD Maastricht, The Netherlands

2 GKC, Maastricht University Medical Centre, 6229 ER Maastricht, The Netherlands

3 Department of Bioengineering and iBB-Institute for Bioengineering and Biosciences, Instituto Superior Técnico, Universidade de Lisboa, Lisboa, Portugal

4 Instituto de Medicina Molecular João Lobo Antunes, Faculdade de Medicina, Universidade de Lisboa, Lisboa, Portugal

5 Associate Laboratory i4HB – Institute for Health and Bioeconomy, Instituto Superior Técnico, Universidade de Lisboa, Lisbon, Portugal

6 Department of Human Biology, NUTRIM, School of Nutrition and Translational Research in Metabolism, Maastricht University Medical Centre, Maastricht, The Netherlands

7 Erasmus MC iPS Facility, Erasmus Medical Center, University Medical Center, Rotterdam, Netherlands

8 Department of Developmental Biology, Erasmus Medical Center, University Medical Center, Rotterdam, Netherlands

9 Department of Psychiatry and Neuropsychology, School for Mental Health and Neuroscience (MHeNs), Faculty of Health, Medicine and Life Sciences, Maastricht University, 6200 MD Maastricht, The Netherlands.

10 Department of Bioinformatics—BiGCaT, Faculty of Health, Medicine and Life Sciences, Maastricht University, 6200 MD Maastricht, The Netherlands.

Correspondence to: Chris P. Reutelingsperger

Email: c.reutelingsperger@maastrichtuniversity.nl

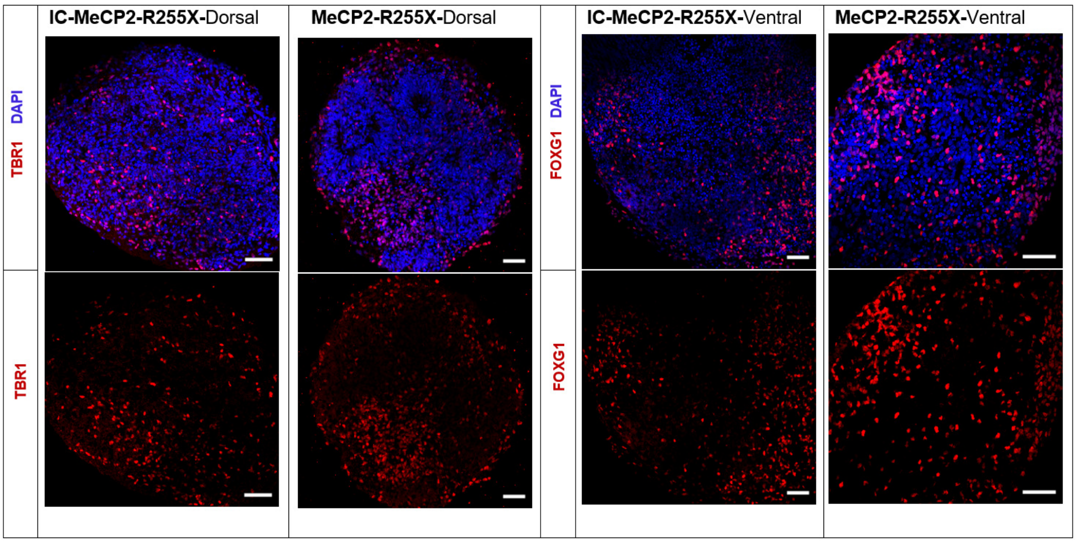

Supplementary Fig. 1 Immunofluorescence characterization of dorsal and ventral organoids at day 41. Representative images of dorsal organoids sections stained against deep cortical layer marker, TBR1 and ventral organoid sections stained against forebrain marker, FOXG1. DAPI was used for nuclear staining. Scale bars, 50 µm. TBR1 is a transcription factor that regulates cortical development, specifically within layer VI of the developing six-layered human cortex. TBR1 is expressed mainly in early-born postmitotic neurons. FOXG1 is a generic forebrain marker, but highly expressed in ventral region.

Tissue preparation was performed according to the protocol by Gomes *et al.* (2020) [1]. Briefly, whole 3D organoids were fixed in 4% PFA for 30 min at 4°C, with agitation, followed by washing in PBS 0.1M. Tissues were incubated overnight in 15% (w/v) sucrose at 4°C.They were then embedded in 7.5% gelatin/15% sucrose and isopenthane (Sigma) which was subsequently used for freezing at −80°C. To prepare organoids section, a cryostat-microtome (Leica CM3050S, Leica Microsystems) was used and sections with approximately 12 µm thickness were collected on Superfrost™ Microscope Slides (Thermo Scientific) (stored at -20 ºC). Organoid sections plated on coverslips were then incubated in 0.1 M Glycine (Millipore) for 10 min at room temperature (RT), followed by permeabilization with 0.1% Triton X-100 (Sigma) for 10 min at RTT. They were then blocked with 10% fetal bovine serum (FBS, Thermofisher Scientific) in TBST [20 mM Tris-HCl pH 8.0, 150 mM NaCl, 0.05% (v/v) Tween-20, Sigma] for 1 h at RT. The cryosectioned organoids were then incubated with primary antibodies, diluted in blocking solution, and incubated overnight at 4°C. Following three washing steps with TBST, they were incubated with secondary antibodies for 45 min at RT. Nuclear counterstaining was performed using 4′,6-diamidino-2-phenylindole (DAPI, 1.5 µg/mL; Sigma). After drying, sections and coverslips were mounted in Mowiol (Sigma). Fluorescence images were acquired using Zeiss LSM 710 Confocal Laser Point-Scanning Microscopes and images were processed in ZEN 2.3 blue edition software (Zeiss). Primary antibodies were TBR1 (rabbit, 1:400, Millipore, AB10554) and FOXG1 (rabbit, 1:400, Cell Signaling Technologies, 29642S). The secondary antibody was goat anti-rabbit IgG and Alexa Fluor®–546 (1:500, Thermofisher Scientific).

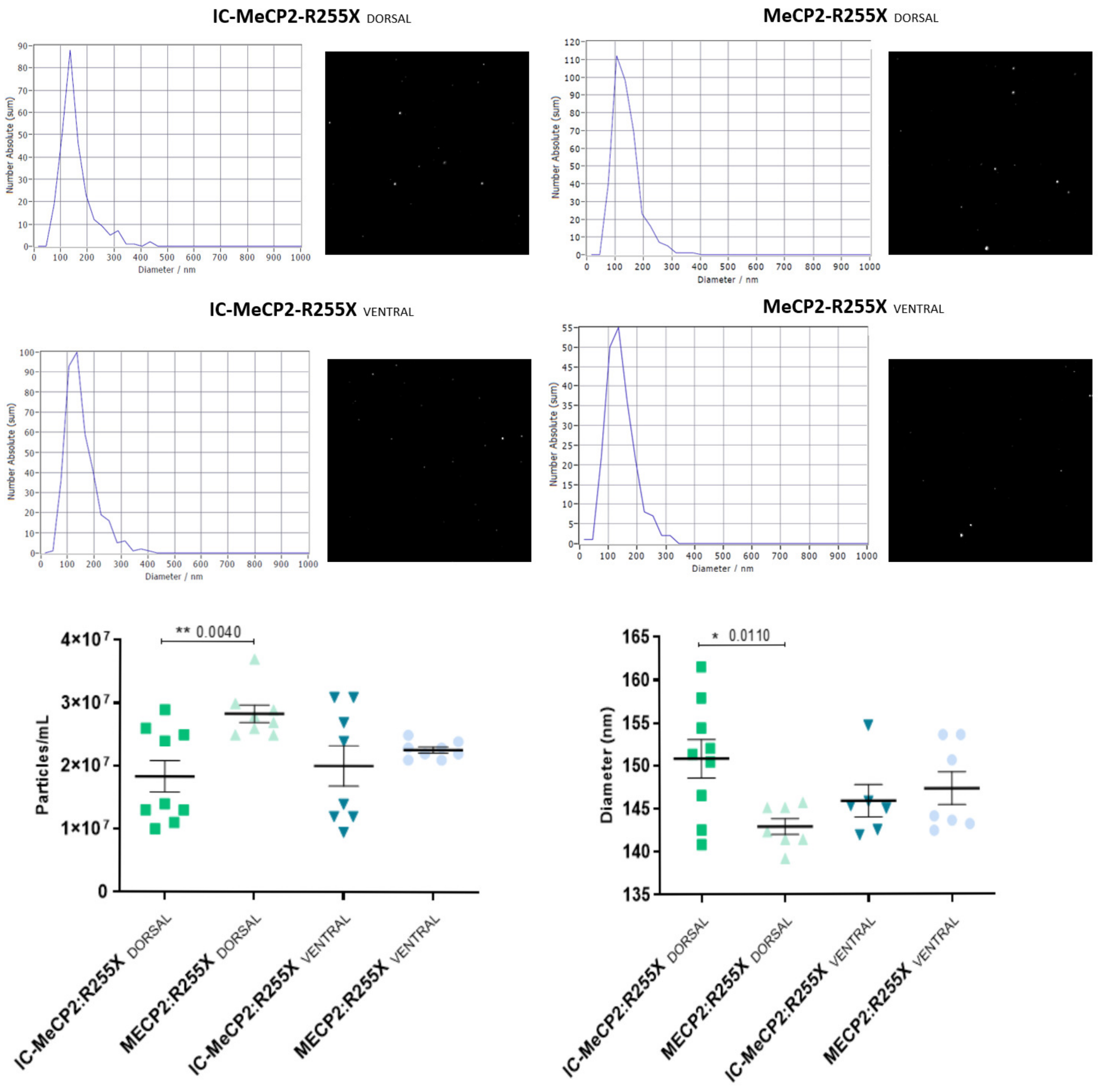

Supplementary Fig. 2 Nanoparticle tracking analysis (NTA) results of EVs size distribution and concentration at day 40. Statistical difference was determined using unpaired two-tailed Student's *t*-test.

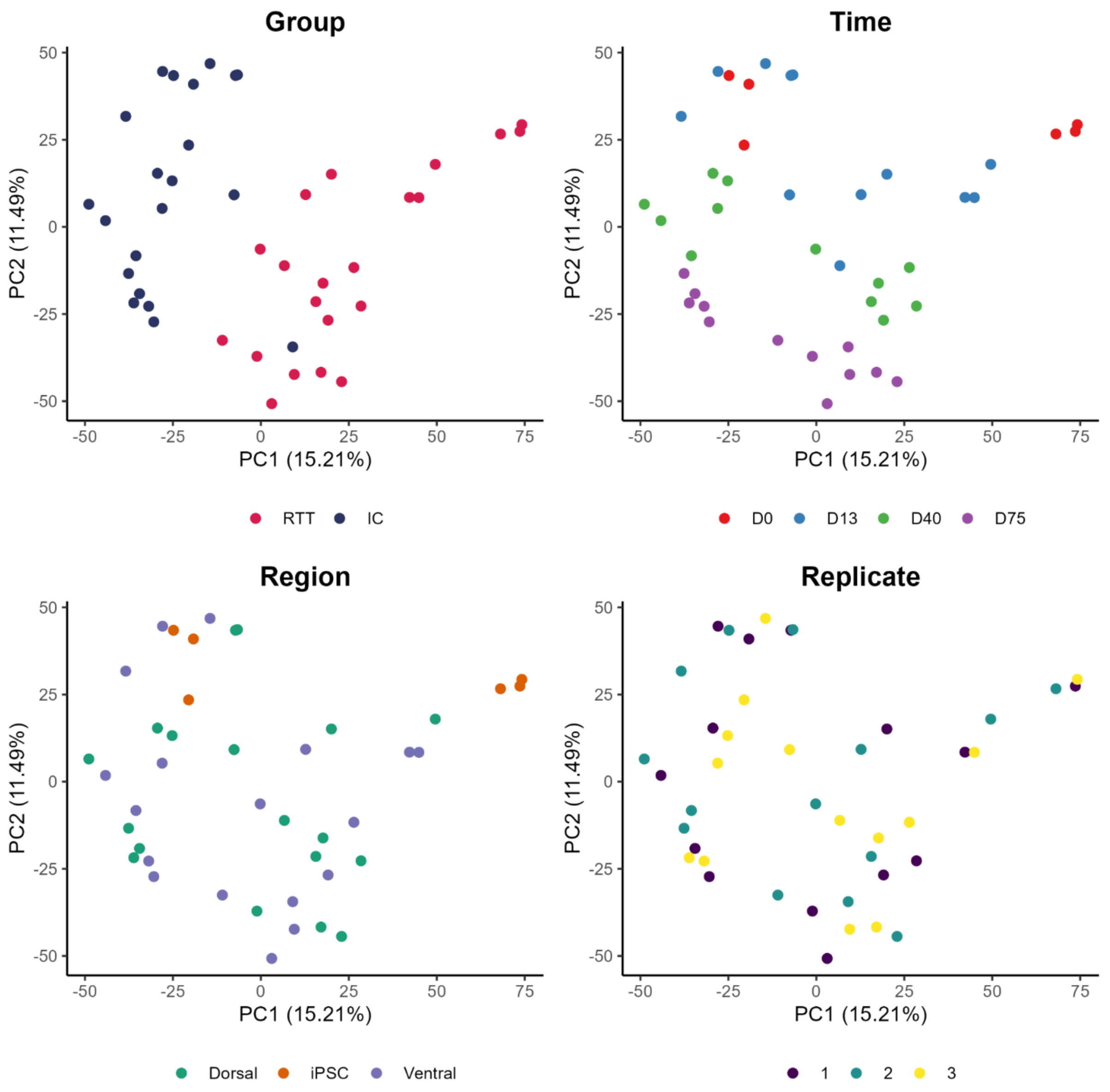

Supplementary Fig. 3 Principal component Analysis (PCA). The post-normalization PCA plot of the first two principal components is colored by the group (top left), time (top right), region (bottom left), and replicate (bottom right) of each sample. Samples are shown to be clustered by their corresponding group and time point.

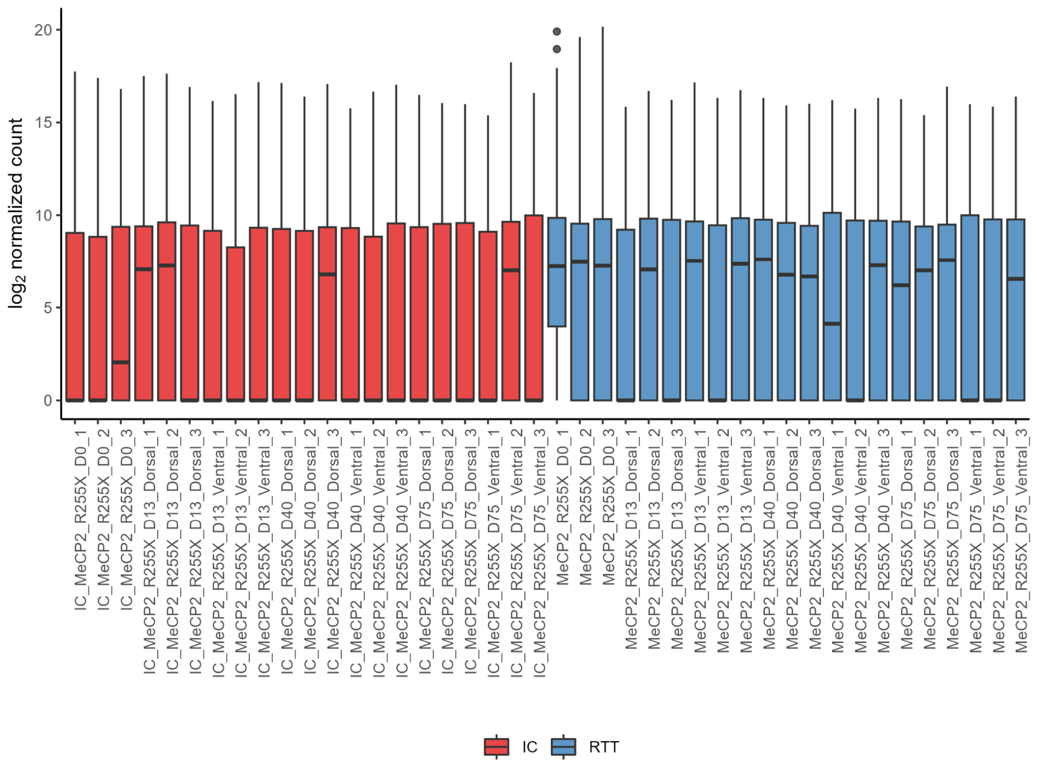

Supplementary Fig. 4 Boxplots of log2 normalized counts. The boxplots show the range of expression values for each sample after the CPM and TMM normalization procedure.

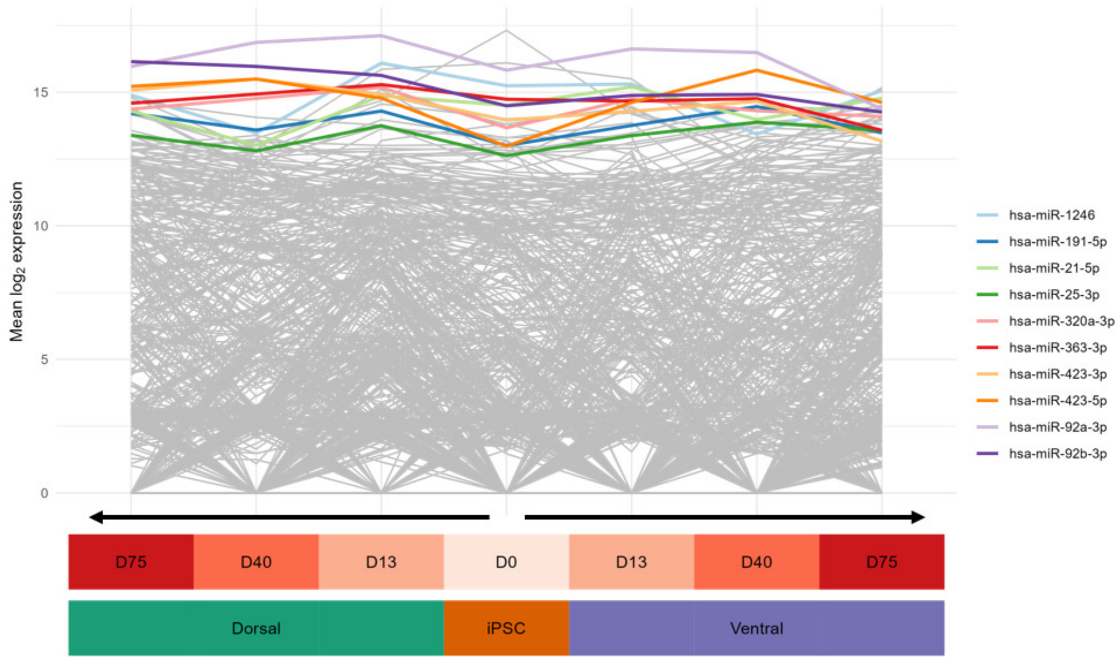

Supplementary Fig. 5 Ubiquitously expressed miRNAs. Mean expression per time point and tissue is shown for miRNAs with a log2 expression of at least 12 in all isogenic control (IC) samples.

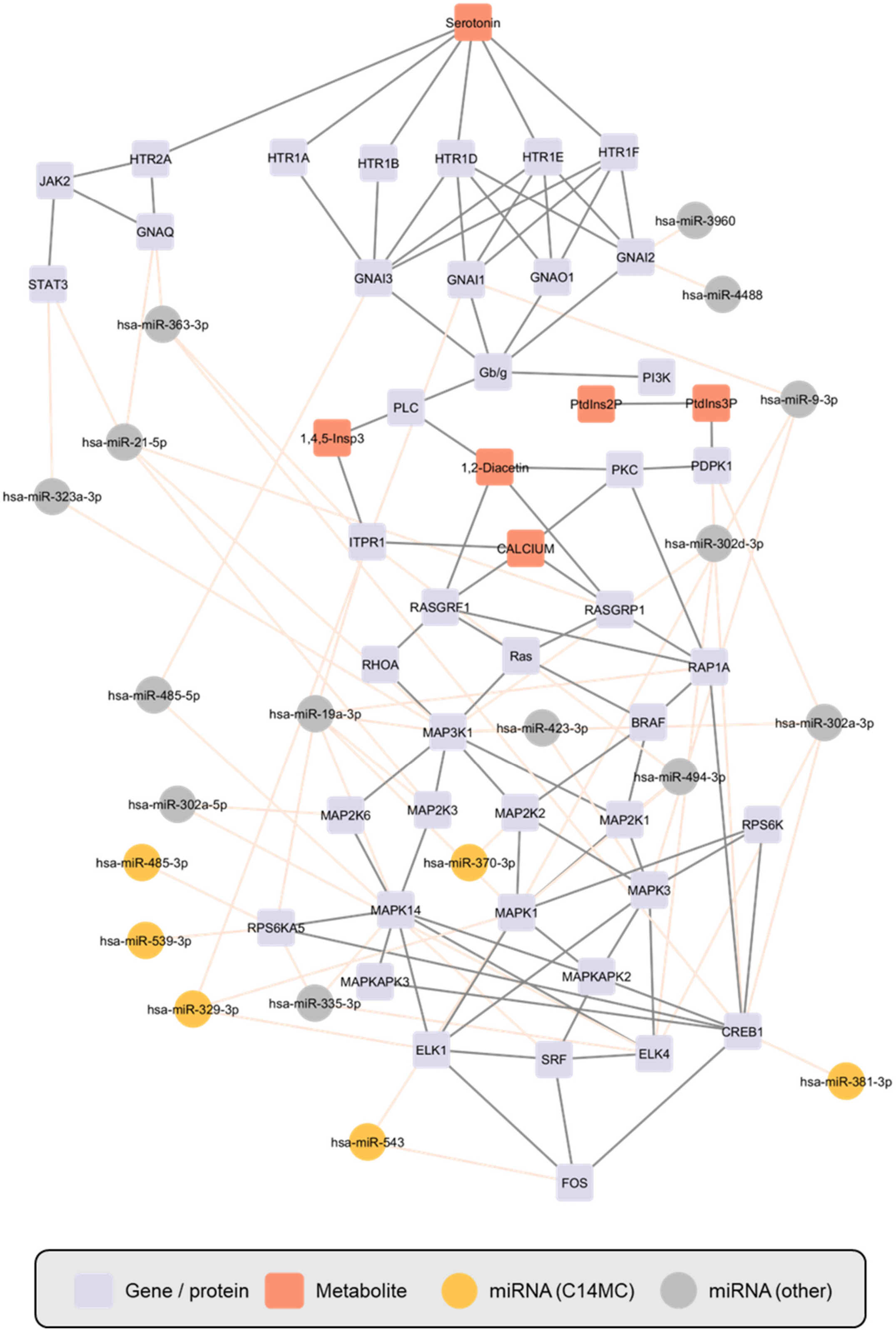

Supplementary Fig. 6 miRNA-target network for the Serotonin HTR1 group and FOS pathway (WP722). The graph shows the gene targets of the significantly differentially expressed miRNAs in the *Serotonin HTR1 group and FOS pathway (WP722)* from WikiPathways.

Supplementary Table 1 Top 30 enriched pathways of miRNAs with a decreasing expression over time. The miRNAs with a decreasing expression profile over time are defined by their membership of the yellow cluster (Fig. 3, main text)

.ID	Description	p-value	FDR
WP61	Notch signaling pathway	5.00E-04	0.281
WP391	Mitochondrial gene expression	0.0026	0.7306
WP2038	Microtubule cytoskeleton regulation	0.0105	0.75096
WP2583	T cell receptor and co-stimulatory signaling	0.0109	0.75096
WP2029	Cell differentiation - index	0.0112	0.75096
WP107	Translation factors	0.0121	0.75096
WP268	Notch signaling	0.0134	0.75096
WP3657	Hematopoietic stem cell gene regulation by GABP alpha/beta complex	0.016	0.75096
WP2815	Mammary gland development pathway - Involution (Stage 4 of 4)	0.0162	0.75096
WP4532	Intraflagellar transport proteins binding to dynein	0.0195	0.75096
WP4290	Metabolic reprogramming in colon cancer	0.0205	0.75096
WP5027	nsp1 from SARS-CoV-2 inhibits translation initiation in the host cell	0.0206	0.75096
WP3871	Valproic acid pathway	0.0218	0.75096
WP1982	Sterol regulatory element-binding proteins (SREBP) signaling	0.0285	0.75096

WP477	Cytoplasmic ribosomal proteins	0.0297	0.75096
WP183	Proteasome degradation	0.0315	0.75096
WP1471	Target of rapamycin signaling	0.0322	0.75096
WP2878	PPAR-alpha pathway	0.0341	0.75096
WP4357	NRF2-ARE regulation	0.0351	0.75096
WP4241	Type 2 papillary renal cell carcinoma	0.0359	0.75096
WP45	G1 to S cell cycle control	0.0369	0.75096
WP2011	SREBF and miR33 in cholesterol and lipid homeostasis	0.0399	0.75096
WP3584	MECP2 and associated Rett syndrome	0.0402	0.75096
WP3414	Initiation of transcription and translation elongation at the HIV-1 LTR	0.0407	0.75096
WP1541	Energy metabolism	0.0413	0.75096
WP5238	Cholestasis	0.0448	0.75096
WP2446	Retinoblastoma gene in cancer	0.0451	0.75096
WP3845	Canonical and non-canonical Notch signaling	0.0457	0.75096
WP179	Cell cycle	0.0513	0.75096
WP2023	Cell differentiation - expanded index	0.0526	0.75096

Supplementary Table 2 Top 30 enriched pathways of miRNAs with an increasing expression over time. The miRNAs with a decreasing expression profile over time are defined by their membership of the pink cluster (Fig. 3, main text)

ID	Description	p-value	FDR
WP4806	EGFR tyrosine kinase inhibitor resistance	3.00E-04	0.058
WP2037	Prolactin signaling pathway	3.00E-04	0.058
WP3935	Leptin-insulin signaling overlap	3.00E-04	0.058
WP4674	Head and neck squamous cell carcinoma	6.00E-04	0.087
WP2453	TCA cycle and deficiency of pyruvate dehydrogenase complex (PDHc)	9.00E-04	0.096667
WP4919	Neuroinflammation	0.001	0.096667
WP2203	Thymic stromal lymphopoietin (TSLP) signaling pathway	0.0013	0.107714
WP78	TCA cycle (aka Krebs or citric acid cycle)	0.0017	0.12325
WP585	Interferon type I signaling pathways	0.0021	0.125667
WP4155	Endometrial cancer	0.0022	0.125667
WP2870	Extracellular vesicle-mediated signaling in recipient cells	0.0026	0.125667
WP1541	Energy metabolism	0.0026	0.125667
WP5083	Neuroinflammation and glutamatergic signaling	0.0031	0.132111
WP4539	Synaptic signaling pathways associated with autism spectrum disorder	0.0033	0.132111

WP2332	Interleukin-11 signaling pathway	0.0039	0.132111
WP2034	Leptin signaling pathway	0.004	0.132111
WP3971	OSX and miRNAs in tooth development	0.004	0.132111
WP395	IL-4 signaling pathway	0.0041	0.132111
WP581	EPO receptor signaling	0.0049	0.134811
WP3414	Initiation of transcription and translation elongation at the HIV-1 LTR	0.005	0.134811
WP2380	Brain-derived neurotrophic factor (BDNF) signaling pathway	0.0055	0.134811
WP2374	Oncostatin M signaling pathway	0.0057	0.134811
WP2261	Glioblastoma signaling pathways	0.0058	0.134811
WP4685	Melanoma	0.0058	0.134811
WP4549	Fragile X syndrome	0.0065	0.134811
WP1544	MicroRNAs in cardiomyocyte hypertrophy	0.0068	0.134811
WP2032	Thyroid stimulating hormone (TSH) signaling pathway	0.0069	0.134811
WP5124	Alzheimer's disease	0.0069	0.134811
WP3872	Regulation of apoptosis by parathyroid hormone-related protein	0.0071	0.134811
WP2849	Hematopoietic stem cell differentiation	0.0076	0.134811

Supplementary Table 3 Top 30 enriched pathways of the ubiquitously expressed miRNAs among all IC samples.

ID	Description	p-value	FDR
WP5027	nsp1 from SARS-CoV-2 inhibits translation initiation in the host cell	6.00E-04	0.345
WP2272	Pathogenic Escherichia coli infection	0.0022	0.4485
WP1541	Energy metabolism	0.0029	0.4485
WP183	Proteasome degradation	0.0032	0.4485
WP391	Mitochondrial gene expression	0.0071	0.4485
WP107	Translation factors	0.0075	0.4485
WP5124	Alzheimer's disease	0.0075	0.4485
WP45	G1 to S cell cycle control	0.0078	0.4485
WP477	Cytoplasmic ribosomal proteins	0.0089	0.4485
WP179	Cell cycle	0.0092	0.4485
WP2059	Alzheimer's disease and miRNA effects	0.0092	0.4485
WP4533	Transcription co-factors SKI and SKIL protein partners	0.0109	0.4485
WP4312	Rett syndrome causing genes	0.0111	0.4485
WP2023	Cell differentiation - expanded index	0.0114	0.4485
WP2267	Synaptic vesicle pathway	0.0117	0.4485
WP3301	MFAP5-mediated ovarian cancer cell motility and invasiveness	0.0127	0.456406

WP3584	MECP2 and associated Rett syndrome	0.0146	0.493824
WP4879	Overlap between signal transduction pathways contributing to LMNA laminopathies	0.0171	0.514118
WP2874	Liver X receptor pathway	0.0177	0.514118
WP4290	Metabolic reprogramming in colon cancer	0.0185	0.514118
WP2882	Nuclear receptors meta-pathway	0.0204	0.514118
WP3959	DNA IR-double strand breaks and cellular response via ATM	0.021	0.514118
WP2359	Parkin-ubiquitin proteasomal system pathway	0.0211	0.514118
WP61	Notch signaling pathway	0.0215	0.514118
WP2446	Retinoblastoma gene in cancer	0.0234	0.514118
WP3995	Prion disease pathway	0.0247	0.514118
WP4204	Tumor suppressor activity of SMARCB1	0.0265	0.514118
WP4241	Type 2 papillary renal cell carcinoma	0.0273	0.514118
WP5050	TCA cycle in senescence	0.0288	0.514118
WP4255	Non-small cell lung cancer	0.029	0.514118

Supplementary Table 4 Top 30 enriched pathways of the differentially expressed miRNAs between RTT and IC.

ID	Description	p-value	FDR
WP722	Serotonin HTR1 group and FOS pathway	0.0016	0.266732
WP3849	MAPK and NFkB signaling pathways inhibited by Yersinia YopJ	0.0018	0.266732
WP4136	Fibrin complement receptor 3 signaling pathway	0.0022	0.266732
WP2795	Cardiac hypertrophic response	0.0024	0.266732
WP1544	MicroRNAs in cardiomyocyte hypertrophy	0.0038	0.266732
WP706	Sudden infant death syndrome (SIDS) susceptibility pathways	0.0038	0.266732
WP2865	IL1 and megakaryocytes in obesity	0.0041	0.266732
WP4262	Breast cancer pathway	0.005	0.266732
WP3879	4-hydroxytamoxifen, dexamethasone, and retinoic acids regulation of p27 expression	0.0058	0.266732
WP5044	Kynurenine pathway and links to cell senescence	0.0061	0.266732
WP4481	Resistin as a regulator of inflammation	0.0063	0.266732
WP1981	Thyroxine (thyroid hormone) production	0.0063	0.266732
WP4787	Osteoblast differentiation and related diseases	0.0067	0.266732
WP400	p38 MAPK signaling pathway	0.0069	0.266732
WP4210	Tryptophan catabolism leading to NAD+ production	0.0071	0.266732

WP2637	Interleukin-1 (IL-1) structural pathway	0.0076	0.266732
WP5192	Modulation of PI3K-Akt-mTOR signaling by bioactive sphingolipids	0.0082	0.266732
WP4396	Nonalcoholic fatty liver disease	0.0086	0.266732
WP195	IL-1 signaling pathway	0.0091	0.266732
WP2533	Glycerophospholipid biosynthetic pathway	0.0097	0.266732
WP4542	Leukocyte-intrinsic Hippo pathway functions	0.0101	0.266732
WP176	Folate metabolism	0.0101	0.266732
WP732	Serotonin receptor 2 and ELK-SRF/GATA4 signaling	0.0117	0.295552
WP585	Interferon type I signaling pathways	0.0127	0.307446
WP75	Toll-like receptor signaling pathway	0.0136	0.316064
WP3676	BDNF-TrkB signaling	0.0143	0.31955
WP2029	Cell differentiation - index	0.0149	0.320626
WP2355	Corticotropin-releasing hormone signaling pathway	0.0161	0.321487
WP3286	Copper homeostasis	0.0162	0.321487
WP272	Blood clotting cascade	0.0166	0.321487

Supplementary References

1 Gomes, A. R. *et al.* Modeling Rett Syndrome With Human Patient-Specific Forebrain Organoids. *Front Cell Dev Biol* **8**, doi:ARTN 610427 10.3389/fcell.2020.610427 (2020).
